# Adipokinetic Hormones Enhance the Efficacy of the Entomopathogenic Fungus *Isaria fumosorosea* in Model and Pest Insects

**DOI:** 10.3390/pathogens9100801

**Published:** 2020-09-28

**Authors:** Umesh Kumar Gautam, Daniela Hlávková, Haq Abdul Shaik, Ismail Karaca, Gürsel Karaca, Kazim Sezen, Dalibor Kodrík

**Affiliations:** 1 Department of Biochemistry and Physiology, Institute of Entomology, Biology Centre, Czech Academy of Sciences, 370 05 České Budějovice, Czech Republic; gautam@entu.cas.cz (U.K.G.); hlavkova.daniela@entu.cas.cz (D.H.); haq@entu.cas.cz (H.A.S.); 2 Department of Zoology, Faculty of Science, University of South Bohemia, 370 05 České Budějovice, Czech Republic; 3 Department of Plant Protection, Faculty of Agriculture, Isparta University of Applied Sciences, 32260 Isparta, Turkey; ismailkaraca@isparta.edu.tr (I.K.); gurselkaraca@isparta.edu.tr (G.K.); 4 Department of Biology, Faculty of Science, Karadeniz Technical University, 61080 Trabzon, Turkey; sezen@ktu.edu.tr

**Keywords:** insect pest, entomopathogen, mortality, AKH, carbon dioxide production, metabolism

## Abstract

Insect adipokinetic hormones (AKHs) are neuropeptides with a wide range of actions, including the control of insect energy metabolism. These hormones are also known to be involved in the insect defence system against toxins and pathogens. In this study, our aim was to demonstrate whether the application of external AKHs significantly enhances the efficacy of the entomopathogenic fungus *Isaria fumosorosea* in a model species (firebug *Pyrrhocoris apterus*) and pest species (Egyptian cotton leafworm *Spodoptera littoralis* and pea aphid *Acyrthosiphon pisum*). It was found that the co-application of *Isaria* with AKHs significantly enhanced insect mortality in comparison to the application of *Isaria* alone. The mode of action probably involves an increase in metabolism that is caused by AKHs (evidenced by the production of carbon dioxide), which accelerates the turnover of *Isaria* toxins produced into the infected insects. However, several species-specific differences probably exist. Intoxication by *Isaria* elicited the stimulation of *Akh* gene expression and synthesis of AKHs. Therefore, all interactions between *Isaria* and AKH actions as well as their impact on insect physiology from a theoretical and practical point of view need to be discussed further.

## 1. Introduction

Entomopathogens are microorganisms (nematodes, fungi and bacteria) that are mostly obligate and lethal for insects and relatively harmless for other animals, including humans. The efficiency of entomopathogens depends not only on their pathogenicity, but also on the insects’ defence reactions and their regulation. However, the interactions between pathogens and insect regulatory endocrine and nervous systems do not seem to have been well researched; this also applies to entomopathogenic fungi (EPFs). Therefore, any findings that can help understand these interactions are considered both theoretically and practically interesting. Generally, EPFs play a significant role in the biological control of pest insects [1] thanks to their worldwide distribution and relatively wide host range. For instance, *Isaria fumosorosea*, *Metarhizium anisopliae* and *Beauveria bassiana* are considered the most promising species because they have been successfully used to control several pest insects [2].

*I. fumosorosea* was isolated for the first time from the sugar beet weevil *Cleonus punctiventris* in Ukraine [3]. Like many other EPFs, *I. fumosorosea* are dimorphic in nature, that is, they exhibit two forms: a mycelial form (asexual non-motile conidium) and a yeast form (vegetative fungus) [4]. The production of conidia starts from conidiogenous cells. These conidia remain dormant in the environment until the conditions are favourable. Attachment of conidia to the exoskeleton of the host stimulates germination, which starts with the production of degrading enzymes, such as proteases, chitinases, chitosanases and lipases [5]. These enzymes, in addition to the development of appressoria (a special pathogenic cell type), allow the formation of hyphal structures that penetrate the cuticle of the host insect. These hyphae reach the host’s haemocoel and produce blastospores, which then undergo budding to allow for rapid propagation and to counteract the host’s immune system [6]. When the host’s haemolymph nutrients are depleted by blastospores, the blastospores start elongating the hyphae, which then produce conidiophores and flask-shaped phialides on the cadaver to complete the cycle of infection.

Both blastospores and conidiospores can be produced artificially in culture media for experimental purposes as well as for pest insect control [7]. Interestingly, the production of blastospores is faster than that of conidiospores, which may help to save time, achieve faster infection, prevent the growth of bacterial contaminants and allow the insect to be infected.

Notably, the spores and mycelial masses of *I. fumosorosea* are not the only factors of pathogenicity; the fungus also produces low-molecular-weight natural compounds. In the last 40 years, more than 20 of such compounds have been identified from *I. fumosorosea* [8], with the most important ones being beauvericin, cepharosporolide C, fumosorinone (an inhibitor of protein tyrosine phosphatase 1B), trichocarane E (carotane-type sesquiterpene) and oxalic acid.

Attacks by entomopathogens are very stressful for the host insects. Thus, it is not surprising that insects have evolved several defence mechanisms to help eliminate or at least reduce the impact of such stress. In insects, under unfavourable conditions, the defensive biochemical and physiological reactions are controlled predominantly by adipokinetic hormones (AKHs). These hormones are members of the AKH/red pigment concentrating hormone arthropod peptide family and are a typical example of neuropeptides with complex functions [9,10]. Generally, AKHs are responsible for the mobilisation of energy substrates from the insect fat body, and they also control a number of processes that complement this main function [9,11,12]. AKH peptides are synthesised and stored in the endocrine glands corpora cardiaca or in the corresponding corpora cardiaca cells of dipteran ring glands from which they are released into the haemolymph when necessary [13].

Generally, AKHs play a role in insect immunity, especially in the defence reactions against insecticides (for a review, see [12,14]), bacterial and fungal toxins [15,16,17,18], nematobacterial complexes [19,20] and insect toxins [21]. Interestingly, it has recently been found that the co-application of these deleterious factors with external AKHs significantly increased mortality in the treated insects [18,19,21,22,23,24]. However, the exact mechanism underlying this effect is still not fully understood. It is thought that the AKH-elicited increase in metabolism may amplify the toxin’s activity through the accelerated metabolite exchange associated with faster toxin penetration into tissues and cells. In the case of living pathogens, the mobilisation of nutrients from the fat body into the haemolymph, mediated by AKHs, could also play an important role.

The main goal of this study was to compare the effects of AKHs on the mortality and metabolism of three insect species infected by the fungus *I. fumosorosea* including one model species (firebug *Pyrrhocoris apterus*) and two pest species (Egyptian cotton leafworm *Spodoptera littoralis* and pea aphid *Acyrthosiphon pisum*). Our aim was also to elucidate the phenomenon in which AKHs increase the mortality of insects infected with pathogens, which might help in controlling pest insect species in the future.

## 2. Results

### 2.1. Effect of Isaria on Mortality

**Figure 1:**
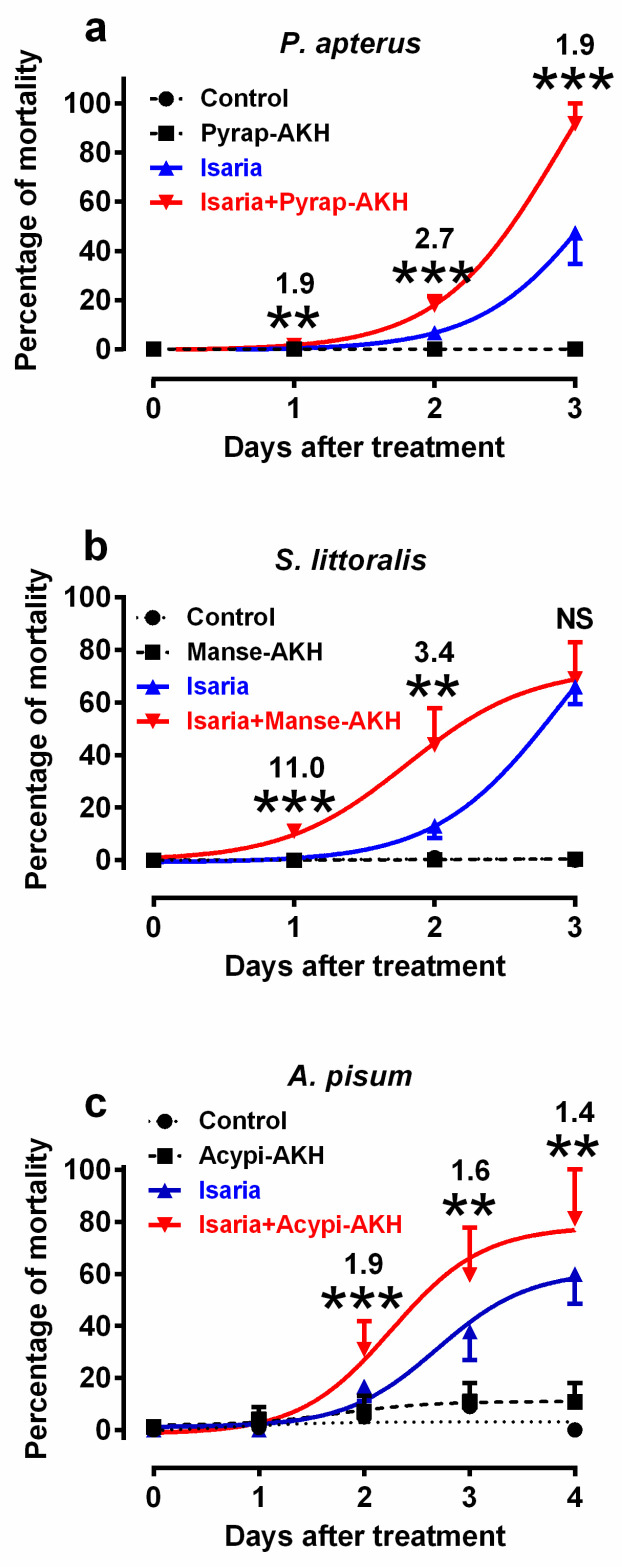
Effect of *Isaria fumosorosea* and adipokinetic hormones (AKHs) on the mortality rates of (**a**) *Pyrrhocoris apterus* (injected by 30,000 blastospores and/or 10 pmol Pyrap-AKH/indiv.), (**b**) *Spodoptera littoralis* (injected by 80,000 blastospores and/or 5 pmol Manse-AKH/indiv.) and (**c**) *Acyrthosiphon pisum* (sprayed by 10^8^ blastospores/mL and/or 3 pmol Acypi-AKH/μL) one to three/four days after treatment. Statistical differences among the curves were evaluated using two-way analysis of variance (ANOVA) with Tukey’s post-test (for detailed results, see Table S1–S6 in the Supplementary Materials). Significant differences between points on *Isaria* and *Isaria* + AKH curves are indicated by * at 5%, ** at 1% and *** at 0.1% levels (*n* = 5–10 groups, with each group comprising 20–30 insects). The numbers above the points represent fold differences between corresponding *Isaria* and *Isaria* + AKH points.

In the first series of experiments, we tested the mortality rates of experimental insects infected with *Isaria* and/or corresponding AKHs. We selected doses of 30,000 and 80,000 blastospores for injection into the bodies of *P. apterus* and *S. littoralis*, respectively, and concentrations of 10^8^ blastospores/mL for topical application to *A. pisum* (Figure 1). These doses were found to elicit optimal mortality within the observation period. Higher and lower doses were not suitable for subsequent physiological experiments as they caused very high and very low mortality, respectively, which would prevent the study of the effect of co-applied hormones. Similarly, optimal hormone doses were determined on the basis of previous studies [25,26,27]. The results revealed that the co-application of AKHs with *Isaria* significantly increased the rate of mortality in comparison to the application of *Isaria* only in all the tested insect species (Figure 1, Table S1–S6). The maximal increase was 2.7-fold in *P. apterus* (two days after treatment, Figure 1a), 11-fold in *S. littoralis* (one day after treatment; Figure 1b) and 1.9-fold in *A. pisum* (two days after treatment; Figure 1c). The mortality rates in the hormonally treated experimental insects or in the Ringer-treated controls were minimal or even null. Statistical differences between these two groups were not significant in *P. apterus* and *S. littoralis,* and were significant, but generally minimal in A. *pisum*; however, this had no effect on the interpretation of the above-mentioned results.

### 2.2. Effect of Isaria on AKH Transcript Abundance

**Figure 2:**
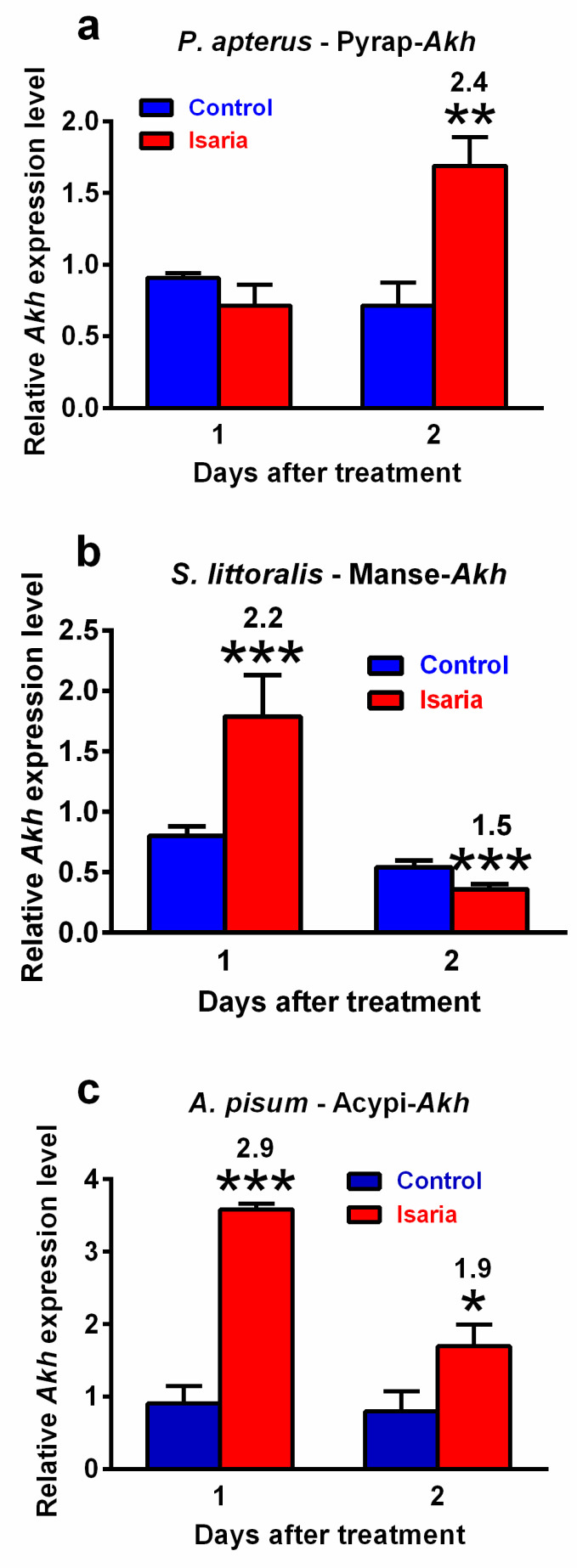
Effect of *Isaria fumosorosea* on *Akh* gene expression in (**a**) *Pyrrhocoris apterus* (injected by 30,000 blastospores/indiv.), (**b**) *Spodoptera littoralis* (injected by 80,000 blastospores/indiv.) and (**c**) *Acyrthosiphon pisum* (sprayed by 10^8^ blastospores/mL) one and two days after treatment. Statistical differences between the *Isaria* groups and corresponding controls were evaluated using Student’s t-test. Significant differences are indicated by * at 5%, ** at 1% and *** at 0.1% levels (*n* = 3). The numbers above the columns represent fold differences between the *Isaria* groups and corresponding controls.

**Figure 3:**
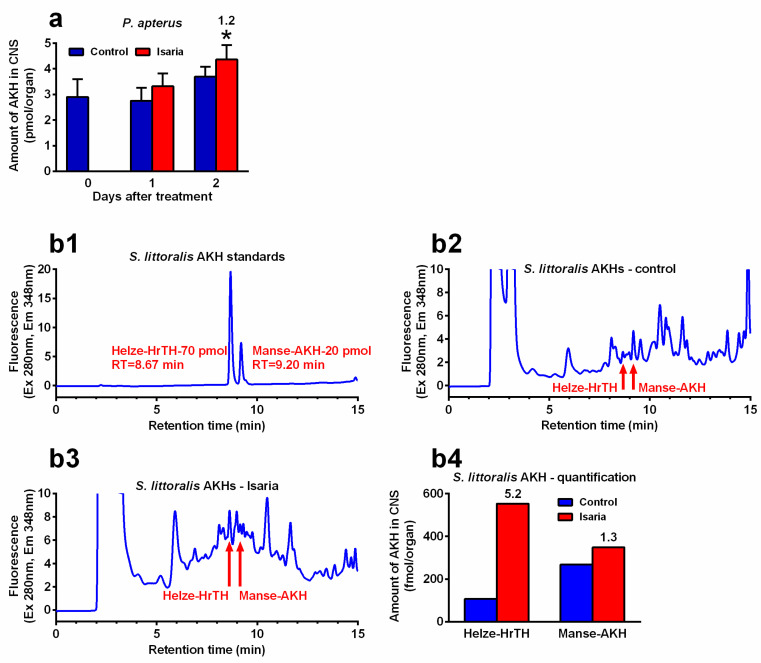
Effect of *Isaria fumosorosea* on the levels of adipokinetic hormones (AKHs) in the central nervous system (CNS) of (**a**) *Pyrrhocoris apterus* (injected by 30,000 blastospores/indiv.) and (**b**) *Spodoptera littoralis* (injected by 80,000 blastospores/indiv.) one to two days (*P. apterus*, (**a**)) and only one day (*S. littoralis*, (**b**)) after treatment. (**b1**–**b3**) show the reversed-phase high-performance liquid chromatography (RP HPLC) profiles: (**b1**) synthetic standards of *S. littoralis* AKHs; (**b2**) methanolic extracts from the CNS of 57 *S. littoralis* individuals treated with Ringer saline (controls); (**b3**) methanolic extracts from the CNS of 57 individuals treated with *I. fumosorosea*; (**b4**) semi-quantification of the results from (**b2**,**b3**). Statistical differences between the *Isaria* groups and corresponding controls (**a**) were evaluated using Student’s t-test. Significant differences are indicated by * at the 5% level (*n* = 6–8). The numbers above the columns represent fold differences between the *Isaria* groups and corresponding controls. RT, retention time.

Infection with *Isaria* elicits severe stress in the treated insects. This stress significantly increased the *Akh* gene expression. The maximal increase was 2.4-fold in *P. apterus* (two days after treatment, Figure 2a), 2.2-fold in *S. littoralis* (one day after treatment, Figure 2b) and 2.9-fold in *A. pisum* (again one day after treatment, Figure 2c). A similar trend was also observed at the peptide level. Using the antibody against Pyrap-AKH (which also recognises the second *P apterus* AKH Peram-CAH-II), we found a slight but significant increase (1.2-fold) in *P. apterus* AKHs in the CNS two days after treatment with *Isaria* (Figure 3a). Semi-quantification of *S. littoralis* AKHs by using reversed-phase high-performance liquid chromatography (RP HPLC) indicated a substantial increase in Helze-HrTH (5.2-fold, Figure 3b) and a slight increase in Manse-AKH (1.3-fold, Figure 3b) one day after treatment. However, reliable quantification of Acypi-AKH in the bodies of *A. pisum* was not successful (see Section 3).

### 2.3. Effect of Isaria on Carbon Dioxide Production and Metabolic Levels

**Figure 4:**
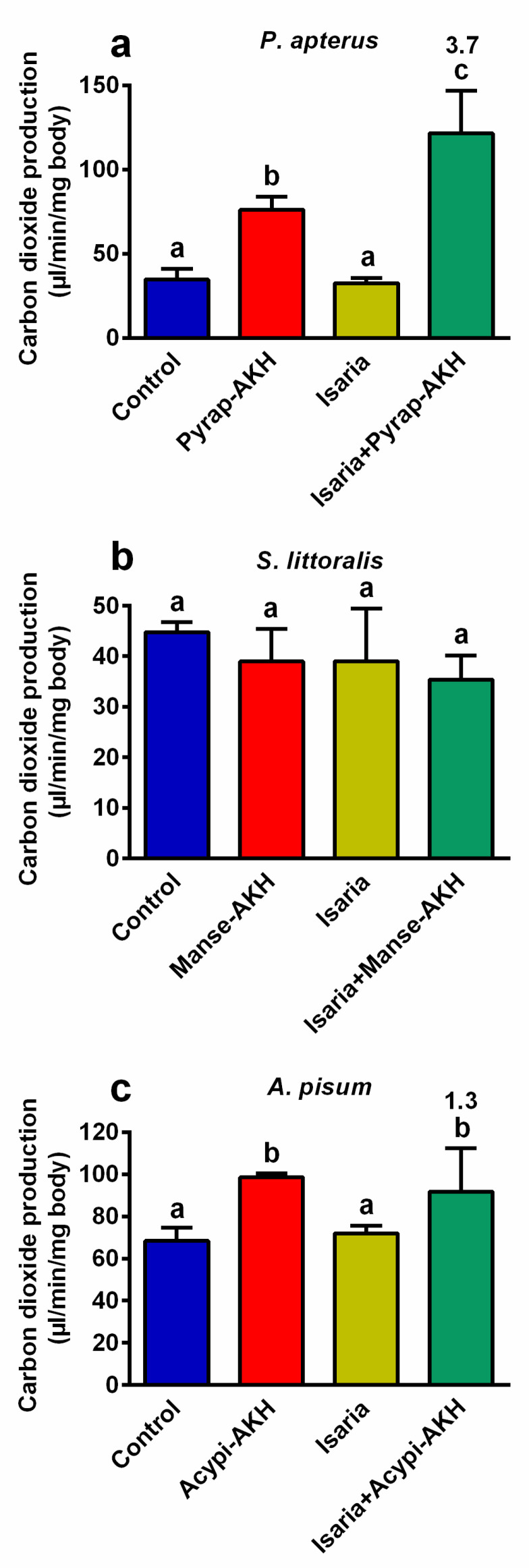
Effect of *Isaria fumosorosea* and adipokinetic hormones (AKHs) on the production of carbon dioxide in (**a**) *Pyrrhocoris apterus* (injected by 30,000 blastospores and/or 10 pmol Pyrap-AKH/indiv.), (**b**) *Spodoptera littoralis* (injected by 80,000 blastospores and/or 5 pmol Manse-AKH/indiv.) and (**c**) *Acyrthosiphon pisum* (sprayed by 10^8^ blastospores/mL and/or 3 pmol Acypi-AKH/μL) one day after treatment. Statistical differences among the groups were evaluated using one-way analysis of variance (ANOVA) with Tukey’s post-test. Significant differences at the 5% level are indicated by different letters (*n* = 6–12). The numbers above the columns represent fold differences between the *Isaria* and *Isaria* + AKH groups.

According to our previous results on the role of AKHs in insects treated with insecticides or entomopathogens [12,14,18,19], we assumed that the increase observed in the metabolic rate due to AKHs played a critical role in the increase in the rate of mortality after co-treatment with *Isaria* and AKHs (Figure 1). Thus, the production of carbon dioxide, which reflects the total level of metabolism, was evaluated. Indeed, carbon dioxide production significantly increased in *P. apterus* (3.7-fold, Figure 4a) and *A. pisum* (1.3-fold, Figure 4b) co-treated with *Isaria* + AKH in comparison to insects treated with *Isaria* alone. In the same two insect species, application of the hormones only also stimulated the production of carbon dioxide, whereas the application of *Isaria* alone had no effect. Interestingly, none of the above-mentioned effects were observed in *S. littoralis*, that is, no significant changes in the production of carbon dioxide were observed.

## 3. Discussion

### 3.1. Effect of Isaria and Externally Applied AKHs on Mortality and Metabolism

Unexpected effects of AKHs have been previously observed in *Locusta migratoria* [28,29], and in *P. apterus* [22]. Co-application of *M. anisopliae* conidia or Gram-positive bacterium *Bacillus megaterium* with AKHs in *L. migratoria*, and co-application of the insecticide permethrin with AKHs in *P. apterus* were found to significantly increase insect mortality in comparison to pathogen/pesticide treatment alone. This AKH effect was confirmed for other insecticides and pathogens in several insect species [14,18,20]. A probable explanation for this counter-productive AKH activity stems from the main role of AKHs, which is the activation of the main energy substrates (lipids, carbohydrates, proline) under metabolic stress [9]. In AKH-treated insects, both metabolism and metabolite turnover are increased. If insecticides or toxins produced by pathogens are present, their turnover might be faster, which may increase their efficacy and their effect on the biochemical and physiological processes in the affected body. Generally, the effect of the application of external AKHs on the mobilisation of nutrients is detectable within a few dozens of minutes (e.g., [30]), which may be faster than the activation of the defence (immune) system [16,18,19,20]. The suggestion that the precise increase in metabolism by AKHs in affected/stressed insects is probably critical was confirmed by measuring the production of carbon dioxide (a marker of metabolic intensity), which becomes significantly higher after AKH co-treatment [14]. Such results were observed in *P. apterus* treated with permethrin [22], endosulfan and malathion [23], as well as in *Tribolium castaneum* treated with pirimiphos-methyl and deltamethrin [24]. Similar results were observed for living pathogens: *P. apterus* and *Drosophila melanogaster* were infected by the entomo-pathogenic nematode *Steinernema carpocapsae* [19,20], and the American cockroach *Periplaneta americana* was infected by *Isaria* [18] before the AKH treatments. In this study, we observed a clear, significant stimulatory effect of AKHs on the rate of metabolism in *P. apterus* and *A. pisum* treated by *Isaria*. Interestingly, the same was not true for *S. littoralis*, in which none of the treatments used changed the rate of carbon dioxide production. This was surprising, especially in relation to the course of the mortality curves, which show patterns in *S. littoralis* that are similar to those observed in other monitored species. It seems that the mechanism of AKH versus *Isaria* action in *S. littoralis* might be different from those studied in insects so far. Although this phenomenon is probably a species-specific one, we cannot suggest a satisfactory alternative at present. Hence, more experiments are needed to explain this.

In the case of living pathogens, the following explanations might help to clarify the role of AKHs in increasing the toxic efficacy. The first was suggested by Mullen and Goldsworthy in 2006 [29], who proposed that the mobilisation of nutrients from the fat body into the haemolymph may facilitate nutrient availability for growing pathogens and, thus, increase their proliferation and virulence. Indeed, we observed this in our recent study while monitoring the effect of *Isaria* and AKHs on the nutrient levels in *P. americana* [18]. We found that the levels of carbohydrates and lipids were approximately 1.8 times higher in the haemolymph of cockroaches treated by *Isaria* + AKH than in those treated by *Isaria* only. Accordingly, the levels of lipids, total free carbohydrates and trehalose were higher in the haemolymph of control *P. apterus* than in those with knocked down expression of the AKH receptor by RNAi, all after treatment by the nematode *S. carpocapsae* [19]. A similar effect was observed in *D. melanogaster* producing non-functional Drome-AKH (AKH^1^; [20]), which lacks a third amino acid in its molecule [31]. These flies infected by the nematode showed a higher trehalose level in control flies than that in AKH^1^ mutant; on the other hand, no significant differences were observed in the lipid levels of these two fly groups.

The second reason why AKHs may stimulate the proliferation of living pathogens may be due to their ability to inhibit protein synthesis. This phenomenon was first described a long time ago by Carlisle and Loughton [32] and later confirmed in various papers. However, so far, it is still not completely known whether AKHs block protein synthesis directly or whether they function via the inhibition of RNA synthesis, as shown in *L. migratoria* [33]. Nevertheless, we cannot exclude the possibility that AKHs reduce the synthesis of anti-microbial peptides. These peptides are generally produced by the fat body cells or haemocytes during the infection of the insect body by pathogens, and they mostly contribute to the defensive immune reaction by disrupting microbial membranes [34].

### 3.2. Effect of Isaria on the Synthesis of Intrinsic AKHs

Stressful conditions increase the levels of AKHs in the haemolymph depending on the intensity and duration of stress in the CNS [11,35]. Treatment of experimental insects by toxins or pathogens represents an ideal model in which the expression of *Akh* genes and the determination of AKH levels can serve as suitable markers. On the other hand, quite high variability has been recorded while monitoring these markers depending on the type of stressor and insect species [14,21,22,35,36]. Nevertheless, a significant increase in the characteristics of AKHs (gene expression, peptide level) was observed after treatment with insecticides or natural toxins in *Schistocerca gregaria* [35], *Leptinotarsa decemlineata* [36] and *P. apterus* [14,21,22]. Similar effects were also observed for entomopathogens: treatment of *P. apterus* by the nematode *S. carpocapsae* elicited a significant increase in *Akh* gene expression as well as an increase in AKH levels in both the haemolymph and CNS [19]. Infestation of *D. melanogaster* larvae by the same nematode exhibited a similar, albeit not so intense, effect [20]. Treatment with *Isaria* also significantly increased both the *Akh* gene expression and AKH levels in the CNS of *P. americana* [18,37]. In this study, we observed significant stimulation of *Akh* gene expression in all the studied insect species after treatment with *Isaria*. These results were clearly manifested by the AKH level in *P. apterus* using anti-Pyrap-AKH antibody and corresponding enzyme-linked immunosorbent assay technique (ELISA). However, obtaining similar quantitative results for *S. littoralis* and *A. pisum* is rather problematic because no antibodies against AKHs from these species are available. Thus, using the semi-quantitative chromatographic approach, there seems to be a strong increase in both Helze-HrTH and Manse-AKH in the CNS of *S. littoralis* after treatment with *Isaria*. However, because of the laboriousness of these analyses (more than 50 CNS samples/run), we could not repeat the experiment to obtain more data for statistical analysis. It still seems that the elevation of Helze-HrTH is more intensive than that of Manse-AKH (about four-fold), which suggests there may be certain specialisation of AKHs. Notably, the phenomenon of possible AKH specialisation in insect species possessing more than one AKH is a problem that has been poorly understood for a long time. Several attempts have been made to solve this problem in the past [33,38,39]. However, despite some promising indications, no satisfactory explanation has yet been provided, nor does this study solve this problem. As mentioned in Section 4, we tried to estimate the level of Acypi-AKH in *A. pisum* using the same methodology that we used for *S. littoralis*. However, despite our intensive efforts, we were not quite sure about the identification of the Acypi-AKH peak using its synthetic analogue; therefore, we did not include the results in this study.

## 4. Materials and Methods

### 4.1. Insects and Their Rearing

Three insect species—the firebug, *Pyrrhocoris apterus* (Heteroptera), Egyptian cotton leafworm, *Spodoptera littoralis* (Lepidoptera), and pea aphid, *Acyrthosiphon pisum* (Homoptera)—were used in this study.

A stock culture of *P. apterus* was reared on linden seeds and water ad libitum under long-day conditions (18 h light:6 h dark) at constant temperature of 26 ± 1 °C. Only 7–10 day-old adult males were used for the experiments (for reasons and other details see our previous papers [22,25]).

The larvae of *S. littoralis* were reared on soybean flour-based semi-artificial Premix diet (Stonefly Industries, Inc., Bryan, TX, USA) and the adults were fed on honey solution under a long day photoperiod (16 h light:8 h dark) at 25 ± 1 °C. One or two day-old last (6th) instar larvae were used for the experiments. They were selected from the colony according to the size and feeding activity.

Aphids *A. pisum* were reared in captive breeding on *Vicia faba* seedlings under a long day photoperiod (16 h light:8 h dark) at 23 ± 1 °C. The last instar of parthenogenetic female nymphs were used for experiments.

### 4.2. Entomopathogenic Fungus Isaria fumosorosea

The fungus *I. fumosorosea* (strain CCM 8367) was used in this study; for its origin see [40,41,42]. We used *I. fumosorosea* blastospores only in this study; for reasons see [18].

### 4.3. Insect Treatment and Dissection

The *I. fumosorosea* blastospores were applied either by injection (*P. apterus*, *S. littoralis*) or topically by spraying (*A. pisum*). In these experiments, doses of 30,000 and 80,000 per individual in 2 μL of Ringer saline were injected into abdominal haemocoel in *P. apterus* and *S. littoralis* individuals, respectively. These doses were determined by initial experiments (data not shown). Corresponding controls were injected with Ringer saline only. In the topical application, the *A. pisum* individuals were sprayed by a suspension containing 10^8^ blastospores per mL Ringer saline (determined by initial experiments; data not shown). The experiments were terminated usually 1 or 2 days later (unless otherwise stated; for mortality data see below).

In some experiments the effect of Pyrap-AKH, Manse-AKH and Acypi-AKH was monitored. These peptides were either prepared in the Polypeptide Laboratories, Prague, Czech Republic (Pyrap-AKH, Acypi-AKH) or purchased from Bachem, Switzerland (Manse-AKH). The hormones were applied according to the same scheme mentioned for the blastospores in the previous paragraph in the following doses/concentrations: 10 pmol Pyrap-AKH, 5 pmol Manse-AKH and 3 pmol/μL Acypi-AKH, respectively. To monitor the effect of *Isaria* and corresponding AKH on the experimental insects, the usual scheme for the experiments was (a) control, (b) application of *Isaria* blastospores, (c) AKH and (d) *Isaria* + AKH together.

To determine mortality, 5–10 groups (each containing 20–30 insects) for each experimental group were monitored 1, 2, 3 or 4 days post treatment.

For dissection of the CNS (brain + corpora cardiaca + corpora allata attached) from *P. apterus* and *S. littoralis*, the insects were first anaesthetised on ice. The dissected CNS were processed immediately. In case of *A. pisum*, whole bodies were used instead.

### 4.4. Expression of Akh Genes

#### 4.4.1. Isolation of RNA and cDNA Synthesis

Total RNAs were extracted from *P. apterus* and *S. littoralis* (CNS), and *A. pisum* (whole body) using RiboZol™ RNA Extraction Reagents (AMRESCO, LLC., Solon, OH, USA) following the manufacturer’s protocol. Further, the obtained RNAs were treated with TURBO DNAfree™ DNase (AMBION^®^ by Life Technologies™, Carlsbad, CA, USA) to purify and avoid contaminant DNA. Reverse transcription was performed using 1 μg of template RNA and random hexamers with the Superscript First-Strand Synthesis System for q-RT-PCR (Invitrogen, Carlsbad, ON, Canada). The following cDNA was amplified through a consequent q-RT-PCR reaction using 1 μL of the RT reaction template and the products were tested on agarose gel electrophoresis.

#### 4.4.2. Quantification of Akh Gene Expressions

Quantification of *Pyrap-Akh, Manse-Akh and Acypi-Akh* genes were performed by q-RT-PCR. No reaction template (NRT) as a control reaction was performed to detect the genomic DNA contamination. Each q-RT-PCR reaction consists of 7 μL SYBR^®^ Premix Ex Taq™II (TaKaRa, Shiga, Japan), 3 μL of 10× diluted cDNA template, 500 nM forward and reverse primers and water in a total volume of 14 μL. For gene specific primers used to amplify Pyrap-*Akh*, Manse-*Akh,* and Acypi-*Akh*, and reference genes see Table S7. The q-RT-PCR reaction was performed on the Light Cycler (Bio-Rad, Hercules, USA) and the program was as follows: initial denaturation step at 95 °C for 3 min, followed by 40 cycles of denaturation at 94 °C for 15 s, annealing at 60 °C for 30 s and elongation at 72 °C for 30 s. A final melt-curve step was included post-PCR (ramping from 65 °C to 95 °C by 0.5 °C every 5 s) to confirm the absence of any non-specific amplification. Four serial dilutions were used to check the efficiency of individual primer pairs by constructing a standard curve. Each parallel group of the q-RT-PCR experiment consists of three independent biological and technical replicates. Bio-Rad CFX Manager software was used to determine the Ct values and reaction efficiency. The Pfaffl [43] method was used to determine the relative gene expression.

### 4.5. Quantification of AKHs

A competitive ELISA was used to determine the total AKH content in *P. apterus* CNS (the antibody, recognising both *P. apterus* AKHs, dilution 1:5000, 0.5 CNS equiv. per well, detection limit 20 fmol per well) according to our previously published protocol [15].

For semi-quantitative estimation of the AKH level in *S. littoralis* CNS, an HPLC system was employed. The dissected CNS were extracted in 80% methanol and after centrifugation the supernatants were evaporated to dryness. Then the pellet was dissolved in 25% solution B (see below) and analysed using HPLC (Waters system, Clarity software, Chromolith Performance RP-18e column 150-4.6 mm (Merck, Darmstat, Germany), fluorescence detection using λ_Ex_ = 280 nm and λ_Em_ = 348 nm wave lengths, gradient from 25 to 87% B in 15 min and a flow rate 1.5 mL/min, solutions A = 0.11% trifluoroacetic acid in water and B = 0.1% trifluoroacetic acid in 60% acetonitrile)—for details see [21].

Retention times of the two *S. littoralis* adipokinetic peptides Helze-HrTH (*Heliothis zea* hypertrehalosaemic hormone; pELTFSSGWGNa) [44,45] and Manse-AKH (*Manduca sexta* AKH; pELTFTSSWGa) [45,46] were identified to be 8.67 min and 9.20 min, respectively, using corresponding synthetic standards. The areas of these standard peaks were used to estimate the AKH level in the experimental samples.

Several attempts were made to estimate the level of Acypi-AKH in *A. pisum*. For that the whole aphid bodies were extracted in 80% methanol and the extracts processed by similar chromatographic procedures as mentioned in the previous paragraph for *S. littoralis*. However, in the resulting HPLC records we were not able to unequivocally recognize the Acypi-AKH peak for semi-quantification. Therefore we had to omit this determination.

### 4.6. Metabolic Rate Determination

The rate of carbon dioxide production by experimental insects was determined using the Li-7000 CO_2_/H_2_O analyser (Li-COR Biosciences, Lincoln, NE, USA) as described previously [22]. Seven individual *P. apterus* males or *S. littoralis* larvae were measured separately in seven measuring chambers 24 h after the treatment for a period of 90 min, with 45 min serving as an accustoming phase and another 45 min as real measurement. For *A. pisum*, 20 last instar female nymphs on a piece of bean leaf were placed into each chamber, and similar pieces of the bean leaves were measured simultaneously. Data were analysed by data acquisition software (Sable Systems, Las Vegas, NV, USA) and the results were calculated according to [47]. For *A. pisum*, the value of carbon dioxide production was reduced by the amount produced by the corresponding bean leaf.

### 4.7. Statistical Analyses and Data Presentation

The results were plotted and statistics were calculated using the graphic software Prism (GraphPad Software, version 6.01, San Diego, CA, USA). Points in the graphs represent mean ± SD, the numbers of replicates (*n*) are depicted in the figure legends. The statistical differences were evaluated by two-way ANOVA with the Tukey’s post-test (mortality), by one-way ANOVA with the Tukey’s post-test (carbon dioxide production) and by Student’s t-test (AKH expression, AKH level).

## 5. Conclusions

In summary, in this paper, we demonstrated that the efficacy of the EPF *I. fumosorosea* in the model species *P. apterus,* and pest species *S. littoralis* and *A. pisum* is significantly enhanced by the application of corresponding AKHs. In *P. apterus* and *A. pisum* the mode of AKH action involves (probably among other unstudied aspects) the intensification of metabolism (as evidenced by the determination of the intensity of carbon dioxide production), which can increase the turnover of *Isaria* toxins produced in the insect body. In contrast, our data suggest that *S. littoralis* exhibits other mechanisms, since no change in carbon dioxide production was observed following AKH or *Isaria* treatments. The stress elicited by *Isaria* treatment on the insect body stimulates the expression of corresponding *Akh* genes and probably the synthesis of AKH molecules. The results of this study are interesting only in theory so far, however, the evidence presented as well as the previous literature referenced indicate that native neuropeptides can enhance infection with pathogens. This might be useful for the future development of environmentally friendly strategies for pest insect control.

## Supplementary Materials

The following are available online at https://www.mdpi.com/2076-0817/9/10/801/s1, Table S1: Statistical details of Figure 1a. Two-way ANOVA multiple comparisons of mortality curves. Tabular results; Table S2: Statistical details of Figure 1a. Two-way ANOVA multiple comparisons of mortality curves. Multiple comparisons; Table S3: Statistical details of Figure 1b. Two-way ANOVA multiple comparisons of mortality curves. Tabular results; Table S4: Statistical details of Figure 1b. Two-way ANOVA multiple comparisons of mortality curves. Multiple comparisons; Table S5: Statistical details of Figure 1c. Two-way ANOVA multiple comparisons of mortality curves. Tabular results; Table S6: Statistical details of Figure 1c. Two-way ANOVA multiple comparisons of mortality curves. Multiple comparisons; Table S7. Primers used for q-RT-PCR.

## References

[1] Meyling N.V., Lübeck M., Buckley E.P., Eilenberg J., Rehner S.A. (2009). Community composition, host range and genetic structure of the fungal entomopathogen *Beauveria* in adjoining agricultural and seminatural habitats. Mol. Ecol..

[2] Zimmermann G. (2008). The entomopathogenic fungi *Isaria farinosa* (formerly *Paecilomyces farinosus*) and the *Isaria fumosorosea* species complex (formerly *Paecilomyces fumosoroseus*): Biology, ecology and use in biological control. Biocontrol Sci. Technol..

[3] Wize M.C. (1904). Die durch Pilze hervorgerufenen Krankheiten des Rübenrusselka fers (*Cleonus punctiventris* Germ.) mit besonderer Berucksichtigung neuer Arten. Bulletin International de l’Académie des Sciences de Cracovie, Classe des Sciences Mathématique et Naturelles.

[4] Deshpande M.V. (1999). Mycopesticide production by fermentation: Potential and challenges. Crit. Rev. Microbiol..

[5] Ali S., Huang Z., Ren S. (2010). Production of cuticle degrading enzymes by *Isaria fumosorosea* and their evaluation as a biocontrol agent against diamondback moth. J. Pest Sci..

[6] Lord J.C., Anderson S., Stanley D.W. (2002). Eicosanoids mediate *Manduca sexta* cellular response to the fungal pathogen *Beauveria bassiana*: A role for the lipoxygenase pathway. Arch. Insect Biochem. Physiol..

[7] Jackson M.A., Payne A.R., Odelson D.A. (2004). Liquid-culture production of blastospores of the bioinsecticidal fungus *Paecilomyces fumosoroseus* using portable fermentation equipment. J. Ind. Microbiol. Biotechnol..

[8] Weng Q., Zhang X., Chen W., Hu Q. (2019). Secondary metabolites and the risks of *Isaria fumosorosea* and *Isaria* farinosa. Molecules.

[9] Gäde G., Hoffmann K.H., Spring J.H. (1997). Hormonal regulation in insects: Facts, gaps, and future directions. Physiol. Rev..

[10] Van der Horst D.J., Van Marrewijk W.J.A., Diederen H.B. (2001). Adipokinetic hormones of insect: Release, signal transduction, and responses. Int. Rev. Cytol..

[11] Kodrík D. (2008). Adipokinetic hormone functions that are not associated with insect flight. Physiol. Entomol..

[12] Kodrík D., Bednářová A., Zemanová M., Krishnan N. (2015). Hormonal regulation of response to oxidative stress in insects—An update. Int. J. Mol. Sci..

[13] Kim S.K., Rulifson E.J. (2004). Conserved mechanisms of glucose sensing and regulation by *Drosophila* corpora cardiaca cells. Nature.

[14] Kodrík D., Plavšin I., Velki M., Stašková T., Montgomery J. (2015). Enhancement of insecticide efficacy by adipokinetic hormones. Insecticides: Occurrence, Global Threats and Ecological Impact.

[15] Goldsworthy G.J., Kodrík D., Comley R., Lightfoot M. (2002). A quantitative study of the adipokinetic hormone of the firebug, *Pyrrhocoris apterus*. J. Insect Physiol..

[16] Goldsworthy G.J., Chandrakant S., Opoku-Ware K. (2003). Adipokinetic hormone enhances nodule formation and phenoloxidase activation in adult locusts injected with bacterial lipopolysaccharide. J. Insect Physiol..

[17] Goldsworthy G.J., Mullen L.M., Opoku-Ware K., Chandrakant S. (2003). Interactions between the endocrine end immune systems in locusts. Physiol. Entomol..

[18] Gautam U.K., Bohatá A., Shaik H.A., Zemek R., Kodrík D. (2020). Adipokinetic hormone promotes infection with entomopathogenic fungus *Isaria fumosorosea* in the cockroach *Periplaneta americana*. Comp. Biochem. Physiol. C.

[19] Ibrahim E., Hejníková M., Shaik H.A., Doležel D., Kodrík D. (2017). Adipokinetic hormone activities in insect body infected by entomopathogenic nematode. J. Insect Physiol..

[20] Ibrahim E., Dobeš P., Kunc M., Hyršl P., Kodrík D. (2018). Adipokinetic hormone and adenosine interfere with nematobacterial infection and locomotion in *Drosophila melanogaster*. J. Insect Physiol..

[21] Shaik H.A., Mishra A., Kodrík D. (2017). Beneficial effect of adipokinetic hormone on neuromuscular paralysis in insect body elicited by braconid wasp venom. Comp. Biochem. Physiol. C.

[22] Kodrík D., Bártů I., Socha R. (2010). Adipokinetic hormone (Pyrap-AKH) enhances the effect of a pyrethroid insecticide against the firebug *Pyrrhocoris apterus*. Pest Manag. Sci..

[23] Velki M., Kodrík D., Večeřa J., Hackenberger B.K., Socha R. (2011). Oxidative stress elicited by insecticides: A role for the adipokinetic hormone. Gen. Comp. Endocrinol..

[24] Plavšin I., Stašková T., Šerý M., Smýkal V., Hackenberger H.K., Kodrík D. (2015). Hormonal enhancement of insecticide efficacy in *Tribolium castaneum*: Oxidative stress and metabolic aspects. Comp. Biochem. Physiol. C.

[25] Kodrík D., Socha R., Šimek P., Zemek R., Goldsworthy G.J. (2000). A new member of the AKH/RPCH family that stimulates locomotory activity in the firebug, *Pyrrhocoris apterus* (Heteroptera). Insect Biochem. Mol. Biol..

[26] Večeřa J., Krishnan N., Mithöfer A., Vogele H., Kodrík D. (2012). Adipokinetic hormone-induced antioxidant response in *Spodoptera littoralis*. Comp. Biochem. Physiol. C.

[27] Jedlička P., Steinbauerová V., Šimek P., Zahradníčková H. (2012). Functional characterization of the adipokinetic hormone in the pea aphid, *Acyrthosiphon pisum*. Comp. Biochem. Physiol..

[28] Goldsworthy G.J., Opoku-Ware K., Mullen L.M. (2005). Adipokinetic hormone and the immune responses of locusts to infection. Ann. N. Y. Acad. Sci..

[29] Mullen L.M., Goldsworthy G.J. (2006). Immune responses of locusts to challenge with the pathogenic fungus *Metarhizium* or high doses of laminarin. J. Insect Physiol..

[30] Kodrík D., Socha R., Zemek R. (2002). Topical application of Pya-AKH stimulates lipid mobilization and locomotion in the flightless bug, *Pyrrhocoris apterus* (L.) (Heteroptera). Physiol. Entomol..

[31] Sajwan S., Sidorov R., Stašková T., Žaloudíková A., Takasu J., Kodrík D., Žurovec M. (2015). Targeted mutagenesis and functional analysis of adipokinetic hormone-encoding gene in *Drosophila*. Insect Biochem. Mol. Biol..

[32] Carlisle J., Loughton B.G. (1979). Adipokinetic hormone inhibits protein synthesis in locusta. Nature.

[33] Kodrík D., Goldsworthy G.J. (1995). Inhibition of RNA synthesis by adipokinetic hormones and brain factor(s) in adult fat body of *Locusta migratoria*. J. Insect Physiol..

[34] Lemaitre B., Hoffmann J. (2007). The host defense of *Drosophila melanogaster*. Annu. Rev. Immunol..

[35] Candy D.J. (2002). Adipokinetic hormones concentrations in the haemolymph of *Schistocerca gregaria*, measured by radioimmunoassay. Insect Biochem. Mol. Biol..

[36] Kodrík D., Krishnan N., Habuštová O. (2007). Is the titer of adipokinetic peptides in *Leptinotarsa decemlineata* fed on genetically modified potatoes increased by oxidative stress?. Peptides.

[37] Karbusová N., Gautam U.K., Kodrík D. (2019). Effect of natural toxins and adipokinetic hormones on the activity of digestive enzymes in the midgut of the cockroach *Periplaneta americana*. Arch. Insect Biochem. Physiol..

[38] Goldsworthy G.J., Davey K.G., Peter R.E., Tobe S.S. (1994). Insect adipokinetic hormones: Are they the insect glucagons. Perspectives in Endocrinology, Proceedings of XII. International Congress of Comparative Endocrinology, Toronto, Canada, 16–21 May 1993.

[39] Kodrík D., Socha R. (2005). The effect of insecticide on adipokinetic hormone titre in insect body. Pest Manag. Sci..

[40] Zemek R., Prenerová E., Weyda F. (2007). The first record of entomopathogenic fungus *Paecilomyces fumosoroseus* (Deuteromycota: Hyphomycetes) on the hibernating pupae of *Cameraria ohridella* (Lepidoptera: Gracillariidae). Entomol. Res..

[41] Prenerová E., Zemek R., Weyda F., Volter L. (2013). Strain of entomopathogenic fungus Isaria fumosorosea CCM 8367 (CCEFO.011.PFR) and the method of controlling insect and mite pests. U.S. Patent.

[42] Prenerová E., Zemek R., Weyda F., Volter L. (2015). Strain of entomopathogenic fungus Isaria fumosorosea CCM 8367 (CCEFO.011.PFR) and the method for controlling insect and mite pests. EPO patent.

[43] Pfaffl M.W. (2001). A new mathematical model for relative quantification in real-time RT-PCR. Nucleic Acids Res..

[44] Jaffe H., Raina A.K., Riley C.T., Fraser B.A., Bird T.G., Tseng C.M., Zhang Y.S., Hayes D.K. (1988). Isolation and primary structure of a neuropeptide hormone from *Heliothis zea* with hypertrehalosemic and adipokinetic activities. Biochem. Biophys. Res. Commun..

[45] Gäde G., Marco H.G., Šimek P., Audsley N., Clark K.D., Weaver R.J. (2008). Predicted versus expressed adipokinetic hormones, and other small peptides from the corpus cardiacum-corpus allatum: A case study with beetles and moths. Peptides.

[46] Ziegler R., Eckart K., Schwarz H., Keller R. (1985). Amino acid sequence of *Manduca sexta* adipokinetic hormone elucidated by combined fast atom bombardment (FAB)/tandem mass spectrometry. Biochem. Biophys. Res. Commun..

[47] Withers P.C. (1977). Measurement of VO2, VCO2 and evaporative water loss with a flow-through mask. J. Appl. Physiol..

