# Supplementary material for: Adipokinetic Hormones Enhance the Efficacy of the Entomopathogenic Fungus Isaria fumosorosea in Model and Pest Insects
Source: Pathogens. 2020 Sep 28;9(10):801. doi: 10.3390/pathogens9100801 (PMC7600585; doi:10.3390/pathogens9100801)
Supplement: Supplementary file 1 [file pathogens-09-00801-s001.pdf]

pathogens

Supplementary Materials

Table S1: Statistical details of Figure 1A. Two-way ANOVA multiple comparisons of mortality curves. Tabular results.

**Source of Variation**	**% of total variation**	**P value**	**P value summary**	**Significant?**	
Interaction	39.63	< 0.0001	****	Yes	
Row Factor	33.13	< 0.0001	****	Yes	
Column Factor	21.34	< 0.0001	****	Yes	
**ANOVA table**	**SS**	**DF**	**MS**	**F (DFn, DFd)**	**P value**
Interaction	28570	9	3174	F (9, 155) = 115.7	P < 0.0001
Row Factor	23886	3	7962	F (3, 155) = 290.3	P < 0.0001
Column Factor	15388	3	5129	F (3, 155) = 187.0	P < 0.0001
Residual	4252	155	27.43		

Table S2: Statistical details of Figure 1A. Two-way ANOVA multiple comparisons of mortality curves. Multiple comparisons.

**Number of Families**	**1**			
**Number of Comparisons Per Family**	6			
**Alpha**	0.05			
**Tukey's multiple Comparisons Test**	**Mean Diff**	**95% CI of Diff**	**Significant?**	**Summary**
Control vs. Pyrap-AKH	0.0	−4.312 to 4.312	No	ns
Control vs. *Isaria*	−13.33	−16.93 to −9.736	Yes	****
Control vs. *Isaria*+Pyrap-AKH	−27.77	−31.71 to −23.84	Yes	****
Pyrap-AKH vs. *Isaria*	−13.33	−16.46 to −10.21	Yes	****
Pyrap-AKH vs. *Isaria*+Pyrap-AKH	−27.77	−31.29 to −24.26	Yes	****
Isaria vs. *Isaria*+Pyrap-AKH	−14.44	−17.03 to −11.86	Yes	****

Table S3: Statistical details of Figure 1B. Two-way ANOVA multiple comparisons of mortality curves. Tabular results.

**Source of Variation**	**% of Total Variation**	**P Value**	**P Value Summary**	**Significant?**	
Interaction	34.04	< 0.0001	****	Yes	
Row Factor	31.27	< 0.0001	****	Yes	
Column Factor	30.66	< 0.0001	****	Yes	
**ANOVA Table**	**SS**	**DF**	**MS**	**F (DFn, DFd)**	**P Value**
Interaction	15478	9	1720	F (9, 64) = 60.08	P < 0.0001
Row Factor	14220	3	4740	F (3, 64) = 165.6	P < 0.0001
Column Factor	13941	3	4647	F (3, 64) = 162.3	P < 0.0001
Residual	1832	64	28.63		

Table S4: Statistical details of Figure 1B. Two-way ANOVA multiple comparisons of mortality curves. Multiple comparisons.

**Number of families**	**1**			
**Number of Comparisons Per Family**	6			
**Alpha**	0.05			
**Tukey's Multiple Comparisons Test**	**Mean Diff**	**95% CI of Diff**	**Significant?**	**Summary**
Control vs. Manse-AKH	0.1000	−4.363 to 4.563	No	ns

Control vs. *Isaria*	−19.50	−23.96 to −15.04	Yes	****
Control vs. *Isaria*+Manse-AKH	−30.75	−35.21 to −26.29	Yes	****
Manse-AKH vs. *Isaria*	−19.60	−24.06 to −15.14	Yes	****
Manse-AKH vs. *Isaria*+Manse-AKH	−30.85	−35.31 to −26.39	Yes	****
Isaria vs. *Isaria*+Manse-AKH	−11.25	−15.71 to −6.787	Yes	****

Table S5: Statistical details of Figure 1C. Two-way ANOVA multiple comparisons of mortality curves. Tabular results.

**Source of Variation**	**% of total variation**	**P value**	**P value summary**	**Significant?**	
Interaction	28.33	< 0.0001	****	Yes	
Row Factor	36.03	< 0.0001	****	Yes	
Column Factor	25.48	< 0.0001	****	Yes	
**ANOVA table**	**SS**	**DF**	**MS**	**F (DFn, DFd)**	**P value**
Interaction	31024	12	2585	F (12. 197) = 45.81	P < 0.0001
Row Factor	39448	4	9862	F (4. 197) = 174.7	P < 0.0001
Column Factor	27905	3	9302	F (3. 197) = 164.8	P < 0.0001
Residual	11118	197	56.44		

Table S6: Statistical details of Figure 1C. Two-way ANOVA multiple comparisons of mortality curves. Multiple comparisons.

**Number of Families**	1			
**Number of Comparisons Per Family**	6			
**Alpha**	0.05			
**Tukey's Multiple Comparisons Test**	**Mean Diff**	**95% CI of Diff**	**Significant?**	**Summary**
Control vs. Acypi-AKH	−4.204	−8.276 to −0.1326	Yes	*
Control vs. *Isaria*	−20.54	−24.69 to −16.38	Yes	****
Control vs. *Isaria*+Acypi-AKH	−31.98	−36.74 to −27.22	Yes	****
Acypi-AKH vs. *Isaria*	−16.33	−19.52 to −13.15	Yes	****
Acypi-AKH vs. *Isaria*+Acypi-AKH	−27.77	−31.72 to −23.83	Yes	****
Isaria vs. *Isaria*+Acypi-AKH	−11.44	−15.47 to −7.410	Yes	****

Table S7. Primers used for q-RT-PCR.

Primer Name	Sequence	Bases	Melting Temp. (°C)	GC Cont. (%)	Product Length
Pyrap-*Akh*-for	GCATCCCAGAGGA CAACTACA	21	64	52.4	
Pyrap-*Akh*-rev	TTTACATTCGTCCTG GGTCA	20	62.9	45	144
Rp49-Ref-for	CCGATATGTAAAAC TGAG AAAC	22	56.9	36.4	-
Rp49-Ref-rev	GGA GCA TGT GCC TGG TCT TTT	21	67.4	52.4	-
Manse-*Akh*-for	TGCGCAGATCACGT TCAG	18	65.2	55.6	
Manse-*Akh*-rev	CGACACAGCCTGGT GAACT	19	64.2	57.9	93
Manse-Act-for	CGAGCGAGAAATC GTGCGTAA	21	69.3	52.4	-
Manse-Act-rev	TGACTTGTCCGTGG GGAAGTT	21	67.8	52.4	-
Acypi-*Akh*-for	TGTTGTTGGCCGTG TTCATGTTG	23	71.8	47.8	
Acypi-*Akh*-rev	AGTGGCTGTTCAAT TCGTGACG	22	68.3	50	209
Rpl-27-for	GCTGTCATAATGAA GACCTACGATGA	26	66	42.3	-
Rpl-27-rev	GGTGAAACCTTGTC TACTGTTACATCT	27	63.4	40.7	-

Note: Pyrap-*Akh* and Acypi-*Akh* primes were obtained from Doležel et al. [1] and Jedlička et al. [2], respectively. Manse-*Akh* gene specific primers were prepared on the basis of conserved regions of related lepidopteran genera and their accuracy was confirmed by sequencing; reference genes were obtained from Večeřa et al. [3].

References

1. Doležel, D.; Šauman, I.; Košťál, V.; Hodková, M. Photoperiodic and food signals control expression pattern of the clock gene, period, in the linden bug, *Pyrrhocoris apterus*. *J. Biol. Rhythms* **2007**, *22*, 335–342.

2. Jedlička, P.; Steinbauerová, V.; Šimek, P.; Zahradníčková, H. Functional characterization of the adipokinetic hormone in the pea aphid, *Acyrthosiphon pisum*. *Comp. Biochem. Physiol.* **2012**, *162*, 51–58.
3. Večeřa, J.; Krishnan, N.; Mithöfer, A.; Vogele, H.; Kodrík, D. Adipokinetic hormone-induced antioxidant response in *Spodoptera littoralis*. *Comp. Biochem. Physiol. C* **2012**, *155*, 389–395.
